# AMRmap: An Interactive Web Platform for Analysis of Antimicrobial Resistance Surveillance Data in Russia

**DOI:** 10.3389/fmicb.2021.620002

**Published:** 2021-03-12

**Authors:** Alexey Y. Kuzmenkov, Ivan V. Trushin, Alina G. Vinogradova, Andrey A. Avramenko, Marina V. Sukhorukova, Surbhi Malhotra-Kumar, Andrey V. Dekhnich, Mikhail V. Edelstein, Roman S. Kozlov

**Affiliations:** ^1^Institute of Antimicrobial Chemotherapy, Smolensk State Medical University of the Ministry of Health of the Russian Federation, Smolensk, Russia; ^2^Department of Medical Microbiology, University of Antwerp, Antwerp, Belgium

**Keywords:** AMRmap, antimicrobial resistance, antimicrobial drugs, surveillance, data analysis

## Abstract

Surveillance of antimicrobial resistance (AMR) is crucial for identifying trends in resistance and developing strategies for prevention and treatment of infections. Globally, AMR surveillance systems differ in terms of organizational principles, comprehensiveness, accessibility, and usability of data presentation. Until recently, the data on AMR in Russia were scarcely available, especially to international community, despite the fact that the large prospective multicenter surveillance in Russia was conducted and data were accumulated for over 20 years. We describe the source of data, structure, and functionality of a new-generation web platform, called AMRmap (https://amrmap.net/), for analysis of AMR surveillance data in Russia. The developed platform currently comprises susceptibility data of >40,000 clinical isolates, and the data on abundance of key resistance determinants, including acquired carbapenemases in gram-negatives, are updated annually with information on >5,000 new isolates. The AMRmap allows smart data filtration by multiple parameters and provides interactive data analysis and visualization tools: MIC and S/I/R distribution plots, time-trends and regression plots, associated resistance plots, prevalence maps, statistical significance graphs, and tables.

## Introduction

The global increase in antimicrobial resistance (AMR) is urging the need for surveillance and monitoring of AMR to guide effective prevention and treatment of infections. Continuous AMR surveillance is essential for identifying emerging threats and assessing the burden of resistance. It provides the necessary ground for disease prevention, rational use of available antibiotics, and development of new antimicrobials, diagnostics, and alternative therapies.

Currently, the results of AMR surveillance studies are most often presented in the form of journal publications. While this traditional way of presentation of data has known advantages, it also has certain limitations. Firstly, there is a natural gap between the time of data collection and presentation to the journal readership, which undermines the relevance of data. Secondly, the fragmentation of data and variation in interpretive criteria applied to antimicrobial susceptibility testing (AST) results hamper their comparison between different studies and publications and assessment of AMR trends over time (Cornaglia et al., 2004; Bedenkov et al., 2016). Recently, these limitations have been addressed, at least partially, by the development of web-based resources for analysis and reporting of AMR surveillance data. Few examples of such platforms include the ECDC Surveillance Atlas of Infectious Diseases that currently hosts the data from the European Antimicrobial Resistance Surveillance Network (ECDC, 2020), the Epidemiological Network (EPI-Net) established under the COMBACTE-MAGNET consortium (COMBACTE-MAGNET, 2020), the CDDEP ResistanceMap (CDDEP, 2020), the Antimicrobial Resistance Patient Safety Atlas by the US Centers for Disease Control and Prevention (CDC, 2020), the British Society for Antimicrobial Chemotherapy (BSAC) Resistance Surveillance Project (Reynolds et al., 2008), the Early Warning and Antimicrobial Resistance Surveillance System (Swedres-Svarm, 2019), the Infectious Diseases Surveillance Information System–Antibiotics Resistance (ISIS-AR) maintained by the Dutch National Institute for Public Health and the Environment (RIVM, 2020), the Japan Nosocomial Infections Surveillance (MHLW, 2000), the Antimicrobial Testing Leadership and Surveillance (ATLAS) system (Pfizer, 2004), the Study For Monitoring Antimicrobial Resistance Trends (SMART) (MSD, 2009), and the SENTRY Antimicrobial Surveillance Program (JMI, 1997). The established AMR surveillance programs differ in terms of organizational principles, data sources, and organism–antibiotic combinations they include. Besides, they provide various options for assessing the AMR data in the form of either annual paginated reports or interactive reports with various degree of flexibility for searching, filtering, and customizing the appearance of the data.

Until now, the data on AMR in Russia was only scarcely available, especially to the international medical community. An effort has been made to build a “nation-wide antibiotic resistance database” by consolidating AMR data from various publications using Internet search, data processing, and machine learning algorithms (Bedenkov et al., 2016). Unsurprisingly, however, the main limitation identified by this approach was the lack of consistency and comprehensiveness and high fragmentation of data coming from various research studies. On the other hand, prospective multicenter surveillance studies on resistance of community and nosocomial bacterial pathogens were conducted by the Institute of Antimicrobial Chemotherapy (IAC) and the Interregional Association for Clinical Microbiology and Antimicrobial Chemotherapy (IACMAC) in Russia since the 1990s until the present, which allowed for building a unique collection of clinical isolates and for accumulating large amount of AMR data from centralized testing (Kozlov et al., 2002; Stratchounski et al., 2005; Stratchounski and Rafalski, 2006; Edelstein et al., 2013; Palagin et al., 2019; Sukhorukova et al., 2019). Here, we report the development of the newer-generation web platform, AMRmap (see text footnote 1), for in-depth analysis and visualization of AMR data collected from prospective microbiological surveillance program in Russia whose functionality extends beyond the limits of the existing online resources.

## Materials and Methods

### Sources of Data

The AMRmap database accumulates the data from prospective multicenter AMR surveillance studies conducted by IAC and IACMAC. In these studies, the central laboratory of IAC annually collects from each participating medical center (hospital) up to 150 consecutive non-duplicate (one of each species per patient/case of infection) clinical isolates together with accompanying case report forms (CRFs) containing anonymous patient clinical and epidemiological data, including source of infection (community- or hospital-acquired, as defined according to standard WHO and CDC criteria) (WHO, 2002; Horan et al., 2008), geographical origin, ward type, site of infection, specimen type, date of collection, and patient age. The specimen types from which the isolates are recovered include blood, primarily sterile body fluids (cerebrospinal, pleural, peritoneal, and synovial fluid), tissue biopsies, urine, bronchoalveolar lavage, endotracheal aspirate, sputum, catheter, prosthetic devices, and other clinically relevant types of specimens. Surface swab specimens, screening, and environmental samples are spared. To avoid preselection bias, any isolates deemed clinically significant are included regardless of their species identity or susceptibility profile determined at a local laboratory. In the central laboratory of IAC, the data recorded in CRFs are checked for consistency by a study coordinator, and the isolates are submitted to mandatory species re-identification, AST, and long-term storage in the collection. The AST of isolates to a wide range of antimicrobial agents is performed by reference methods (agar dilution to fosfomycin and broth microdilution to all other antibiotics) according to ISO 20776 and EUCAST methodology (ISO 20776-2:2007,2007; ISO 20776-1:2019,2019; EUCAST, 2020b). The susceptibility categories to antimicrobial agents are determined and updated according to current EUCAST clinical breakpoints for minimum inhibitory concentrations (MICs) (EUCAST, 2020a). Based on resistance phenotypes, selected isolates are subjected to molecular detection of important genetic resistance determinants (e.g., all Enterobacterales, *Pseudomonas*, and *Acinetobacter* isolates with reduced susceptibility to carbapenems are tested by real-time PCR for acquired carbapenemase genes according to EUCAST recommendations) (EUCAST, 2017). Besides the data on resistance markers and susceptibilities of prospectively collected isolates, the AMRmap database also contains the data on the isolates referred to IAC laboratory from various hospitals across Russia and neighbor countries, Belarus and Kazakhstan, for confirmation of exceptional and epidemiologically important resistance phenotypes and genotypes. The data on the latter isolates are included in the absolute figures in the “Genetic Resistance Determinants” section of the website but not in the percentage prevalence figures to avoid bias.

### Statistical Data Analysis and Programming Tools

Analysis of data is performed using the following statistical methods: calculation of absolute and relative frequencies, median values, confidence intervals by Wilson method, multiple comparisons using Fisher’s exact test with Holm’s correction, graph algorithms for visualization of multiple comparisons, and kernel regression for trend analysis.

The web platform is developed using the R programming language and software environment for statistical computing (R Core Team, 2018) and makes use of the following packages: “shiny” for interactive web interface (Chang et al., 2020), “ggplot2” for graphics (Wickham et al., 2020), “data table” and “DT” for aggregating data in tables (Dowle et al., 2020; Xie et al., 2020), “visNetwork” for network visualization (Almende et al., 2019), “leaflet” for geographical maps (Cheng et al., 2019), and “highcharter” for wrapping Highcharts JavaScript graphics library and modules (Kunst et al., 2020).

## Results

### Data Access, Selection, and Filtration

At the time of submission of this paper, the AMRmap website (see text footnote 1) comprised information on susceptibilities to more than 50 antimicrobial agents and combinations of 41,999 clinical isolates, including members of the following groups: Enterobacterales (*n* = 16,390), *Staphylococcus* spp. (*n* = 6,578), *Pseudomonas* spp. (*n* = 5,569), *Acinetobacter* spp. (*n* = 3,778), *Streptococcus pneumoniae* (*n* = 2,701), *Enterococcus* spp. (*n* = 2,155), *Streptococcus* groups A, B, C, G (*n* = 2,092), *Haemophilus* spp. (*n* = 1,026), *Stenotrophomonas maltophilia* (*n* = 660), and *Helicobacter pylori* (*n* = 276) collected in 59 cities of the Russian Federation over the period of 2000–2018. The AMRmap database is updated annually with information on more than 5,000 newly collected isolates.

The main page of AMRmap provides two options for accessing the data: (i) open “public” access that allows assessment of aggregated data from national and regional level down to the level of cities and (ii) personalized “expert” access granted to the coordinator and participants of the AMR surveillance studies that enables analysis and comparison of the data down to the level of individual hospitals (e.g., comparison of the local and regional resistance prevalence).

**FIGURE 1:**
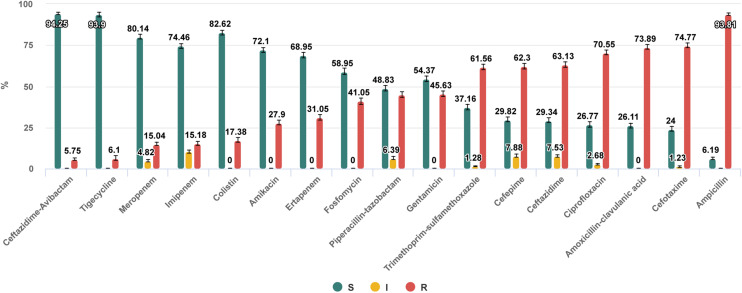
Antibiotic susceptibilities of nosocomial Enterobacterales isolates collected in 53 hospitals, 30 cities of Russia in 2017–2018. https://amrmap.net/? id=AAGZM10rI45rI10.

**FIGURE 2:**
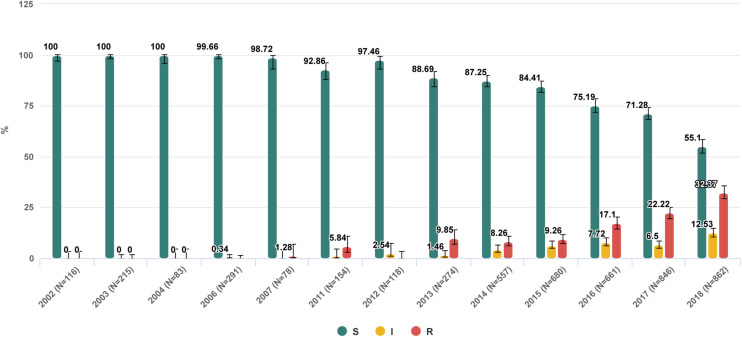
Trend of nosocomial *Klebsiella pneumoniae* resistance to meropenem in Russia from 2002 to 2018. https://amrmap.net/?id=AZv5d51eA44eA11.

**FIGURE 3:**
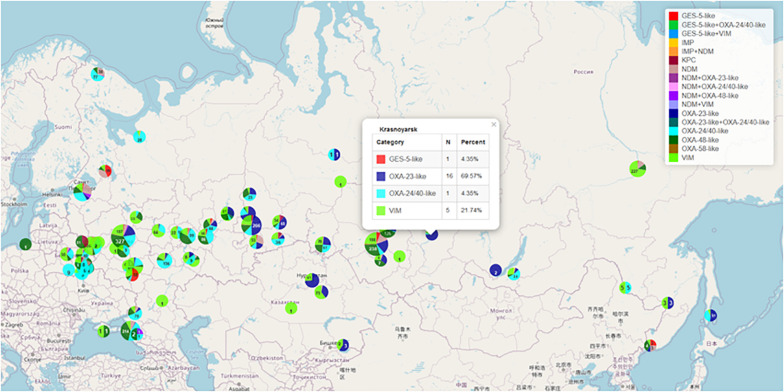
Geographical distribution of gram-negative bacteria producing various types of acquired carbapenemases. https://amrmap.net/?id= oDavO19uA00uA11.

The upper part of the “Data analysis” page contains the block of parameters used to select and filter the data according to group of infections (community- or hospital-acquired or any of the above), geographical origin (federal district, region, city, and institution), department type, site of infection, specimen type, time period, patient age, and microorganism group and species. The filters are organized hierarchically such that the selection of values for one parameter affects available options for the others (for example, selection of “Skin and Soft Tissue” for the site of infection limits the choice of available specimen types to “Abscess,” “Biopsy,” “Blood,” and “Wound Discharge”). All text filters support selection of multiple values and quick text searching. The time and age scales support free selection of ranges at a 1 year discretion. The user can hide and restore the panel of filters by pressing the “∨” button at the top-right corner of the screen. Most importantly, by pressing the nearby “Link” button, the user creates a permanent URL link and a QR code to selected parameters and related infographics (chart, table, or map) displayed on the screen that can be shared with the other users or inserted as a reference for citing the results of analysis. Examples of such analysis results with the corresponding web links are shown in Table 1 and Figures 1–3.

**TABLE 1:** Examples of surveillance data presented on the AMRmap website.

Data example	Interpretation	AMRmap analytics section and subsection	Web links
The most frequently isolated groups and species of nosocomial bacterial pathogens in Russia (2017–2018).	The gram-negative bacteria: Enterobacterales (51.9%), *Pseudomonas* spp. (16.9%), and *Acinetobacter* spp. (15.3%) dominate the epidemiology of nosocomial infections in Russia.	Organisms	https://amrmap.net/?id=4UhlM08Go23Go11
Antibiotic susceptibilities of nosocomial Enterobacterales (2017–2018).	The data indicate a high prevalence of resistance to antibiotics commonly used for treatment of nosocomial infections, including oxyimino-cephalosporins (62.4–74.9%) and carbapenems (15.1–31.3%).	Antibiotic SIR Summary, Plot	https://amrmap.net/?id=AAGZM10rI45rI10
Prevalence of resistance to meropenem in nosocomial *Klebsiella pneumoniae* by year.	Between 2006 and 2018, the resistance to meropenem has increased exponentially and was significantly higher in 2018 than in any preceding year (32.6%; 95%CI 29.5–35.8%; *p* < 0.05)	Selected Antibiotic, Plot by	https://amrmap.net/?id=AZv5d51eA44eA11
Geographic prevalence of methicillin (oxacillin) resistance in *Staphylococcus aureus* (2017–2018).	The map (4A) shows a considerable geographic variation in MRSA prevalence (0–66.7%) with median prevalence in different regions of 16.1% (4B).	Selected Antibiotic, Map, Rating	4A: https://amrmap.net/?id=lm2IM59nP47nP15 4B: https://amrmap.net/?id=agmOk52NP16NP07
MIC distribution (5A) and time-trend MIC distribution (5B) of oxacillin against *Staphylococcus* spp.	The MIC distribution of oxacillin against clinical isolates of staphylococci remains fairly stable over time with MIC50 and MIC90 values corresponding to 0.5 and 256 mg/L, respectively.	Selected Antibiotic, MIC, MIC Trend	5A: https://amrmap.net/?id=CUEHW04nt42nt08 5B: https://amrmap.net/?id=I7Oxx36Ln42Ln08
Associated resistance rates to various antibiotics in nosocomial *Acinetobacter baumannii* (2017–2018).	Carbapenem-resistant *A. baumannii* isolates exhibit extremely high rates of associated resistance to all non-beta-lactam antibiotics except colistin (1.6%), trimethoprim-sulfamethoxazole (40%), and tobramycin (69%) (6A). A pan-resistant phenotype is observed in 0.7% (95% CI: 0.34–1.43) of the isolates (6B).	Associated Resistance, Matrix, Multiple resistance	6A: https://amrmap.net/?id=ah5t124yd50yd09 6B: https://amrmap.net/?id=F3K1N442L512L09
Geographical distribution of gram-negative bacteria producing various types of acquired carbapenemases.	The map (7A) shows the distribution of various acquired carbapenemases in Enterobacterales, *Pseudomonas*, and *Acinetobacter* spp. in various regions of Russia, Belarus, and Kazakhstan. The most prevalent carbapenemases in Russia are the OXA-48-like and NDM in Enterobacterales (7B), VIM and GES-5-like in *Pseudomonas* spp. (7C), and OXA-24/40- and OXA-23-like in *Acinetobacter* spp. (7D).	Genetic Resistance Determinants, Map, Markers	7A: https://amrmap.net/?id=oDavO19uA00uA11 7B: https://amrmap.net/?id=VmpKA506P166P11 7C: https://amrmap.net/?id=Qza0M39mr17mr13 7D: https://amrmap.net/?id=7kLJD24Ll19Ll11
Percentage of nosocomial Enterobacterales carrying the genes of different acquired carbapenemases by year.	The data show a steady increase in the proportion of nosocomial Enterobacterales carrying the acquired carbapenemase genes from 0% in 2010 to 27.7% in 2018.	Genetic Resistance Determinants, Trend, Relative	https://amrmap.net/?id=Uu36C16rI28rI15
Susceptibility of carbapenemase-producing Enterobacterales (CPE) isolates to various antibiotics.	Colistin and ceftazidime-avibactam are the most active *in vitro* against CPE (82.4% and 75% susceptible isolates). About 30% of CPE are categorized as “susceptible” and another 16% are categorized as “susceptible, increased exposure” to imipenem and meropenem with the current EUCAST breakpoints.	Genetic Resistance Determinants, SIR Summary	https://amrmap.net/?id=0cN8I35N441N407
MIC distribution of imipenem (10A) and meropenem (10B) for Enterobacterales carrying the genes of different carbapenemases.	*In vitro* MICs of carbapenems are generally high for isolates carrying the genes of NDM and KPC carbapenemases than those of OXA-48-like carbapenemases.	Genetic Resistance Determinants, MIC	10A: https://amrmap.net/?id=R0sXI22Cj08Cj11 10B: https://amrmap.net/?id=zxYXu09YZ09YZ11
Comparison of meropenem resistance rates among nosocomial and community *Pseudomonas aeruginosa* isolates (2017–2018).	Resistance to meropenem is more than five times higher in nosocomial than in community *P. aeruginosa* isolates (49.4% vs. 9.1%, *p* = 0.0001)	Comparison, Summary	https://amrmap.net/?id=ug9Ob08im21im11
Comparison of ciprofloxacin resistance rates among nosocomial and community urinary *Escherichia coli* isolates (2015–2016).	Resistance to ciprofloxacin is two times higher in nosocomial than in community urinary *E. coli* isolates (63.6% vs. 32%, *p* = 0.0001)	Comparison, Summary	https://amrmap.net/?id=nTRiC44ae26ae11

### Interactive Data Analysis

The block of analytics located below the block of parameters contains six modules (sections): “Organisms,” “Antibiotics SIR Summary,” “Selected Antibiotic,” “Associated Resistance,” “Genetic Resistance Determinants,” and “Comparison” each including subsections and various visualization tools arranged in separate tabs. In all sections, the interactive features allow the user to customize the appearance of charts, maps, and tables. For example, where applicable, the user can select the option of showing separate “Susceptible (S),” “Increased exposure susceptible (I),” and “Resistant (R)” categories or merging the “S” and “I” categories into one category, “S + I.” Another option allows the user to set the maximum allowable 95% confidence interval (CI) for data points to be displayed on the plots and, thus, to screen off the results of low statistical significance (e.g., where the number of isolates is too small to make conclusive evidence). The control elements allow saving charts as vector or raster graphics, saving geographical maps as HTML files with preserved interactive controls (zooming, panning, and opening pop-up infoboxes), and saving tables as text (CSV), Excel, or PDF files.

The “Organisms” section contains the data on species distribution of microorganisms matching the selected parameters. By clicking the “Display TOP 10” button, the user is presented with an interactive chart showing the most frequently isolated species and groups of organisms (Example 1 in Table 1).

The “Antibiotics SIR Summary” section provides the multiple antibiotic susceptibility report in the form of interactive bar chart and in tabular format detailing for each antibiotic the percentage and 95% CI values of S, I, and R isolates (Example 2 in Table 1 and Figure 1).

The “Selected antibiotic” section contains a series of visualization tools to gain detailed information on *in vitro* activity of a particular antibiotic with regard to the prevalence, dynamics, geographical patterns of resistance, and the MIC distribution of isolates. The user can sub-stratify the results by various categories including the time period, group of infections, geographical origin of isolates, department type, site of infection, specimen type, and organism species. The results can be displayed as stacked-bar, bar-chart, or trend-line plots with 95% CI, and the statistical significance of differences between the categories can be assessed using the graph or matrix plot below the main chart (Example 3 in Table 1 and Figure 2).

The “Map” subsection provides access to interactive visualizations of geographical S/I/R prevalence data. There are several map types the user can select from: the classical choropleth map, which uses a range of color shades to represent the susceptibility or resistance rates by geographical region; the multiple-layer map, which uses alert symbols colored green or red according to the defined threshold of resistance rate; and the pie chart map featuring the S/I/R prevalence pie charts positioned over the respective geographic points (locations). The latter map type is displayed by default with the size of the pie charts shown in proportion to the number of isolates representing each geographic point (Example 4 in Table 1). The user can zoom and pan the map to change views, open the map in full-screen mode, and choose between various map styles. The “Regression” graph shows the distribution of various cities along the calculated temporal trend line of resistance prevalence. The “Rating” and “Tree” tabs provide yet more detailed statistics on geographical variability and rating of cities, regions, and federal districts by resistance rates. The interactive features of the map and charts allow users to customize their appearance and open popups or tables with contextual data. For example, selecting any area with data points on a regression graph opens the table with statistical data on the respective cities.

In addition to categorical S/I/R data analysis, the assessment of quantitative resistance data is available under the “MIC” subsection. Standard MIC distribution and time-trend MIC distribution can be displayed for any organism–antibiotic combinations, including, in some cases, those for which no clinical breakpoints have been defined by EUCAST. Elements of the MIC charts are colored green, yellow, and red according to S, I, and R susceptibility categories, or colored gray if interpretive criteria do not exist for a particular organism–antibiotic combination or if the same MIC value corresponds to different categories depending on species ID of isolates. By dragging the mouse cursor over a certain area of “MIC-trend” chart and highlighting MIC data points, the user can display the epidemiological information on the corresponding isolates in the table under the chart (Example 5 in Table 1).

The “Associated resistance” section provides the tools to assess the prevalence of associated resistance to any antibiotics. The results are presented in various forms, including the square “Matrix” with values in each cell indicating the percentage of isolates resistant to antibiotic in a column out of the total number of isolates resistant to antibiotic in a row (Example 6 in Table 1); the “Table” that can be browsed for any combination of antibiotics; and the “Trend” chart that shows the rate of associated resistance to selected antibiotics over time. Other useful analytical instruments included in this section are the “Multiple resistance” calculator for estimating the percentage of isolates simultaneously resistant to selected antibiotics and the “MIC Scatter plot” for analyzing the relative MIC distributions of any antibiotics.

The “Genetic Resistance Determinants” section comprises the data on geospatial and temporal distribution of the clinically and epidemiologically important genetic resistance markers in certain groups of pathogens. Currently, these include the following: genes of acquired carbapenemases of VIM, IMP, NDM, KPC, GES-2/5, OXA-48, OXA-23, OXA-24/40, and OXA-58 groups in >8,000 clinical isolates of the order Enterobacterales and the genera *Acinetobacter* and *Pseudomonas* collected in Russia and neighbor countries of Belarus and Kazakhstan; genes of mobile colistin resistance (*mcr*) in clinical isolates of Enterobacterales; and mutations associated with resistance to macrolides and fluoroquinolones in *Mycoplasma genitalium* and *Mycoplasma pneumoniae*. The geographic distribution and prevalence of selected genetic markers can be seen in the “Map” subsection (Example 7 in Table 1 and Figure 3).

Temporal distributions of selected resistance markers can be seen under the “Trend” subsection. The “Absolute” trend displays, by year, the absolute number of isolates carrying selected resistance determinants including the isolates collected in the frame of prospective surveillance program and those referred to the reference laboratory for confirmation of resistance mechanisms, while the “Relative” trend shows the percentage prevalence of resistance markers among the isolates collected as part of prospective surveillance only (Example 8 in Table 1).

Importantly, the “Genetic Resistance Determinants” section also provides information about antibiotic susceptibilities of isolates harboring specific genetic markers. In the “SIR Summary” subsection, the user can view the aggregate susceptibility data to various antibiotics, and in the “MIC” subsection, the user can view the detailed information on MIC distribution of any antibiotic according to selected genetic markers (Examples 9 and 10 in Table 1).

The “Comparison” section, as implied by the name, allows the user to compare any datasets (groups of isolates) defined by the two sets of filters with regard to prevalence of resistance (“Summary” and “By Year” subsections), MIC distributions of any antibiotics (“MIC” subsection), and prevalence of resistance determinants (“Determinants” subsection). The results of comparisons are displayed as interactive bar charts depicting the percentage of isolates, 95% CI, and statistical significance *p*-values (Examples 11 and 12 in Table 1).

## Discussion

This paper describes a new web resource^1^ that provides access to the Russian national AMR surveillance data. Unlike many existing regional, national, and international surveillance systems that aggregate routine AST data from local microbiological laboratories, the described surveillance system comprises information obtained from centralized testing of microbial isolates received from participating laboratories across Russia. Each local laboratory annually contributes a defined number of consecutive non-duplicate clinical isolates (one isolate of each species per patient/case of infection) recovered from various types of specimens: blood, other sterile body fluids, tissue biopsies, urine, lower respiratory tract specimens, etc., and accompanied by CRFs detailing basic patient demographics and characteristics of infection. Non-clinical, “screening” and environmental isolates are not included in the surveillance. The central laboratory then confirms the identification of the isolates, determines the MICs of antibiotics by reference methods, and undertakes further testing, including the detection of important genetic resistance determinants and molecular subtyping of selected pathogens.

The described AMR surveillance and reporting system resembles in design the well-established systems built upon the prospective collection and centralized testing of microbial isolates, most notably, the BSAC Resistance Surveillance Project (Reynolds et al., 2008) and the SENTRY Antimicrobial Surveillance Program (JMI, 1997). The high quality and consistency of data ensured by the use of common protocols and reference methods in a central laboratory are certainly the main advantages of a prospective surveillance program. The accumulation of standardized MIC, rather than just S/I/R categories, which are subject to change over time, allows for the monitoring of evolutionary trends of resistance. The multicenter design and inclusion of consecutive non-duplicate isolates limit the impact of selection bias and over-representation of outbreak isolates and isolates with specific resistance traits (Cornaglia et al., 2004). Furthermore, the large collection of isolates built over time from a centralized surveillance together with extensive information on resistance profiles of isolates forms a unique resource for further research.

With much similarity to the other programs, the Russian AMR surveillance has some distinctive features. Firstly, it is not restricted to any particular type of infection or organism–antimicrobial combinations, nor does it set the limits for the number of isolates of each species collected annually. This allows, at least an approximate, estimation of species distribution in different types of infections and patient populations and allows for the accumulation of AMR data on common, not-so-common, and emerging pathogens alike (e.g., *S. maltophilia* in nosocomial infections).

Secondly, there is a mandatory stratification of all collected isolates and infections they represent between community- and hospital-acquired. This stratification is made at local centers based on commonly accepted criteria (WHO, 2002; Horan et al., 2008). It is important to note that, according to current practices in Russia, microbiological analysis is more often performed in the cases of severe infections, ineffective primary or empirical therapy, and hospital-acquired infections, as reflected in the high proportion of nosocomial isolates (62.5%) in the AMRmap database. Examples 11 and 12 in Table 1 clearly demonstrate that the antibiotic resistance rates are significantly higher in nosocomial than in community isolates. This should be taken into consideration, especially when comparing the AMR data from Russian surveillance and those from other national and international programs. Therefore, the AMRmap interface allows selection, in the top-priority filter, of the hospital- or community-acquired infections, or both for separate or combined analysis of species distribution and AMR data.

Thirdly, the AMRmap web platform offers unmatched flexibility in terms of data filtration, analysis capabilities, and representation. To our knowledge, this is the only open web resource that allows, in addition to common analysis of prevalence and trends of resistance, sub-stratification of isolates by any characteristics and statistical comparison of groups with respect to prevalence of phenotypic resistance and resistance determinants. Other unique features of AMRmap analytics include quantitative assessment of associated resistance to any antibiotics, regression trends with analysis of outlying data points, plots of MIC change over time, and MIC distributions of antibiotics according to genetic determinants. The AMRmap visualization and reporting tools are designed in accordance with the European Centre for Disease Prevention and Control Guidelines for presentation of surveillance data (ECDC, 2018) and, meanwhile, they offer high degree of flexibility and customization. For example, the geographical map style can be changed between classical choropleth map, symbol map, or pie chart map, with the latter, in our opinion, being the most accurate and informative for presenting the geographical patterns of phenotypic resistance and genetic markers in large geographic areas with uneven distribution of population and samples.

At the same time, our surveillance system has certain limitations, the main limitations being the relatively low population coverage and lack of population denominators. These limitations are intrinsic to the programs that involve the collection of microbial isolates for centralized testing rather than of routine AST data (Cornaglia et al., 2004). Nevertheless, our surveillance program has been continuously expanded to include more collecting centers throughout Russia. In 2018–2020, 95 centers, mostly large urban hospitals, from 44 cities in all federal districts of Russia took part in the surveillance, and we hope this number to increase in the future with the inclusion of smaller hospitals from more geographic locations.

The importance of inclusion in our surveillance of isolates from diverse infection sites, besides blood, is echoed by the recent report from the BSAC surveillance program that highlights the disparity in resistance rates among certain bloodstream and respiratory pathogens (Horner et al., 2020). Nonetheless, the low proportion of blood isolates (9% in the AMRmap database) is certainly another limitation of our surveillance, which reflects the low utilization of blood culture diagnostics in Russia (WHO/Europe, 2020) and leaves room for improvement in the future.

Currently, more than 100 microbiologists and clinicians from surveillance centers across Russia have registered access to the AMRmap website, which provides them with detailed information about local AMR. The open-access AMR data available on the website is actively used in the development of hospital and community antimicrobial formularies and treatment guidelines. According to Google Analytics, the entire audience of the AMRmap exceeds 17,000 active users in Russia and, in total, 20,000 users in 108 countries worldwide. We, therefore, conclude that the AMRmap represents a valuable source of information and provides unique capabilities for in-depth analysis of AMR of community and nosocomial pathogens in Russia.

## Data Availability Statement

All relevant data is contained within the article. The original contributions presented in the study are included in the Table 1, further inquiries can be directed to the corresponding author.

## Author Contributions

AK and IT participated in a discussion about how to improve data collection and about the features needed in the web system. ME wrote the initial draft of the manuscript. AD, AV, and RK contributed to the conception and provided critical revision of the intellectual content of the manuscript. AA and MS created the figures and the table. ME, AV, and SM-K were involved in the manuscript preparation. AK and RK supervised the project. All authors contributed to the article and approved the submitted version.

## Conflict of Interest

The authors declare that the research was conducted in the absence of any commercial or financial relationships that could be construed as a potential conflict of interest.

## Footnotes

1 www.amrmap.net
